# Depression-related difficulties disengaging from negative faces are associated with sustained attention to negative feedback during social evaluation and predict stress recovery

**DOI:** 10.1371/journal.pone.0175040

**Published:** 2017-03-31

**Authors:** Alvaro Sanchez, Nuria Romero, Rudi De Raedt

**Affiliations:** Department of Experimental Clinical and Health Psychology, Ghent University, Ghent, Belgium; University of Akron, UNITED STATES

## Abstract

The present study aimed to clarify: 1) the presence of depression-related attention bias related to a social stressor, 2) its association with depression-related attention biases as measured under standard conditions, and 3) their association with impaired stress recovery in depression. A sample of 39 participants reporting a broad range of depression levels completed a standard eye-tracking paradigm in which they had to engage/disengage their gaze with/from emotional faces. Participants then underwent a stress induction (i.e., giving a speech), in which their eye movements to false emotional feedback were measured, and stress reactivity and recovery were assessed. Depression level was associated with longer times to engage/disengage attention with/from negative faces under standard conditions and with sustained attention to negative feedback during the speech. These depression-related biases were associated and mediated the association between depression level and self-reported stress recovery, predicting lower recovery from stress after giving the speech.

## Introduction

According to cognitive models [1–3], depression is caused and maintained by biases in the processing of emotional information. A wealth of empirical research has provided evidence for depression-related emotional biases in visual attention processes. Former studies using attention allocation paradigms, such as the dot-probe task [4] or the spatial cueing task [5] have found that depressed people tend to allocate attention disproportionally more to negative compared with positive or neutral material (i.e., self-descriptive adjectives, facial expressions), but only under conditions of long stimuli exposures (see [6, 7]). These results led to speculate that depressed individuals may not direct their attention to negative information more quickly than do control participants, but once it captures their attention they may exhibit difficulties disengaging from it (e.g., [8]). In recent research, eye-tracking technology has been used to delineate the time course and components of attention biases in depression, showing that, relative to controls, depressed individuals show an increased maintenance of gaze on negative stimuli when they are attended (see [9]).

Although previous eye-tracking studies support the presence of a prolonged processing of negative information in depression, the hypotheses that sustained eye-gaze would reflect impairments in volitional disengagement of attention has remained unclear until the development of direct ways to index such attention patterns. A new eye-tracking paradigm, the attentional engagement-disengagement task [10], has been developed in order to examine the time to disengage attention from emotional faces (happy, angry and sad) when having to engage attention with neutral faces of the same person. Using this paradigm, it has been found that, compared to healthy controls, clinically depressed participants took longer to move their gaze from sad faces towards neutral faces when prompted to [10]. This attention bias is thought to be associated with a lack of inhibitory control over negative information and with the use of maladaptive emotion regulation strategies [6], which would result in sustained negative affect. In order to test this assumption, the predictive role of this depression-related attention bias in self-reported stress reactivity and recovery from a relevant stress task (anticipating giving a speech) has been examined. Consistently, individual differences in attentional disengagement from negative faces predicted lower mood recovery after the stress anticipation in the clinically depressed group [10]. These findings have helped to increase our understanding of the cognitive mechanism involved in negative mood maintenance in depression. However, further steps require clarifying the specific nature of the effect and the conditions under which those mechanisms operate.

### Depression-related attention biases under socio-evaluative situations

One core feature of depression is the experience of difficulties in social interactions [11], which leads to sustained negative affect and post-event rumination [12]. A hypothesis derived from current eye-tracking evidence is that the observed attention biases for emotional faces in depression may be at the basis of those difficulties. Emotional faces comprise salient features of the social environment [13]. Therefore, if attention of depressed individuals is biased to sustain the processing of negative facial expressions [9], as a result of difficulties disengaging from them [10], this may affect how social situations are interpreted and thereby affect emotional responding and the adequate selection of regulation strategies [14]. However, although previous research has demonstrated the presence of depression-related attention biases during standard conditions of emotional faces viewing, it remains unclear whether such biases would also emerge during relevant socio-evaluative situations.

In order to investigate this research question, new paradigms have recently been developed to assess attentional processes during socio-evaluative situations (i.e. an impromptu speech). Participants view a pre-recorded video, where the audience has been trained to display positive, negative or neutral expressions, and participants are told that the audience is online listening to his/her speech. Using eye-tracking to monitor gaze towards the pre-recorded audience during the speech it has been found that healthy individuals are characterized by longer times attending to positive feedback (i.e., positive expressions) than to other types of social feedback [15]. In contrast, participants showing shuttering problems [15] and high social anxiety levels [16] are characterized by longer fixation durations on negative feedback (i.e., negative expressions). Whether a similar behavioral pattern during the processing of socio-evaluative feedback may characterize depression, as the result of observed attentional disengagement impairments, remains untested. The present study aimed to clarify this question, by using a modification of the original eye-tracking based impromptu speech paradigm. Whereas the original paradigm uses a static feedback audience (positive, negative and neutral audience members appears in the same proportion on the screen and do not change across the whole speech duration), we aimed to test attention to a socio-evaluative feedback in dynamic change, as natural social interactions involve dynamic rather than static audiences [17]. This was achieved with a controlled-paradigm where pictures representing the feedback audience varied in the type of feedback depicted (e.g., from moments representing balanced positive-negative feedback to moments representing clearly rejecting negative feedback) across the speech.

Using this paradigm, our first aim was to test the hypothesis of an involvement of depression-related attention biases in maladaptive processing during socio-evaluative situations. We had two different predictions. First, if depression is characterized by maladaptive attentional processes during social interactions, depressive symptoms will be related to sustained processing of negative over positive feedback during the socio-evaluative situation, as observed in other problems related with difficulties in social interactions [15, 16]. Second, if, as proposed, depression-related attention biases are at the basis of maladaptive attentional processes during social interactions, patterns of sustained processing on negative feedback during the socio-evaluative situation should be associated with difficulties in the attentional disengagement from negative facial expressions as measured under standard conditions in former research [10]. In order to test these two predictions, participants first completed the attention engagement-disengagement task [10] followed by the impromptu speech paradigm. This design allowed to test: 1) whether higher depressive symptom severity levels are associated with longer sustained attention to negative over positive feedback during the socio-evaluative situation, and 2) whether such biased attention patterns during the socio-evaluative situation are associated to depression-related attention biases indexed under standard conditions of processing (i.e., longer times to disengage attention from negative faces, as measured in the attentional engagement-disengagement task).

### Depression-related attention biases and stress regulation

Our second aim was to replicate and extend previous evidence on the role of depression-related attention biases (i.e., difficulties in attentional disengagement from negative faces under standard conditions; longer sustained attention to negative over positive feedback under a stressful socio-evaluative condition) in stress regulation. Differences in these attention processes are thought to facilitate the generation of sustained negative affective conditions in depression, as the result of impaired stress regulation [14]. Specifically, difficulties disengaging attention from negative faces in depression have been found to be associated with impaired mood regulation after anticipating a stressful situation (i.e., giving a speech; [10]). Similarly, recent evidence using the impromptu speech combined with eye-tracking has shown that the total duration of fixations on negative feedback during the speech was predictive of subjective anxiety ratings immediately after it [16].

Whereas previous evidence in the context of depression refers to associations between attention biases to social information and subjective mood state changes in response to social stress (self-reported mood states: [10]), the predictive role of these biases has not yet been tested using objective physiological indicators of stress reactivity and recovery. Previous evidence suggests that maladaptive attention bias might be specifically involved in subsequent inefficient regulation of the induced stress responses [10], which would reflect diminished parasympathetic activity to inhibit initial sympathetic influences associated to the stress response [18]. Stressor-induced suppression of cardiac parasympathetic activity [19] has been documented in a growing number of studies using heart rate variability (HRV) as an indirect measure of parasympathetic (vagal) control over time-related variations in heart rate (e.g., [20]). HRV reflects an objective indicator of individual differences in regulating emotional conditions or cognitive functions [21–23]. Therefore, in the present study HRV was used as a physiological measure of parasympathetic activity of stress regulation, during the speech task as well as during a recovery period afterwards. We predicted that depression-related attention biases would be associated with lower subjective self-reported mood recovery (i.e., inefficient stress regulation; [10]), and with lower HRV (i.e. less parasympathetic control) after confrontation with the socio-evaluative situation.

## Materials and methods

### Participants

Individuals with minimal to severe depressive symptoms were sampled from the Ghent University research participant pool based on a prescreening measure (Mood and Anxiety Symptom Questionnaire; [24]). At testing, 39 participants (36 female; age range: 18–36) reported a broad range of depressive symptom severity levels (range: 0–42, *M* = 11.28, *SD* = 10.50) on the Beck Depression Inventory-II ([25]; Dutch translation: [26]), with 25 individuals reporting minimal (20: 0–9; 5: 10–13), 7 mild (14–19), 4 moderate (20–28), and 3 severe symptom levels (29–63). All participants were native Dutch speakers with normal or corrected-to-normal vision. They provided written informed consent and were paid 15 euro for their participation. The study was approved by the faculty ethical committee at Ghent University.

### Questionnaires

#### Depressive symptom severity

The BDI-II assessed depressive symptom severity. On 21 items rated on a four-point scale, respondents indicated the extent to which they suffered from depressive symptoms in the past two weeks. This measure has good reliability and validity in both healthy and depressed samples [25, 26]. The internal consistency in this study was α = .95.

#### Mood state

Mood ratings were administered using three visual analogue scales (VAS), providing measures of happiness, sadness and tension to evaluate mood states at different times across the experimental session. Participants were asked to describe how they felt ‘at that moment’ by indicating on horizontal 100 cm lines whether they experienced the three above-mentioned mood states, from ‘ totally not’ to ‘very much’.

#### Other self-reported measures

Other questionnaires administered in the study for exploratory reasons but not used to test the current hypotheses were the State-Trait Anxiety Inventory (STAI; [27]), the Fear of Negative Evaluation Scale (FNE; [28]), the Ruminative Responses Scale (RRS; [29]) and the Cognitive Emotion Regulation Questionnaire (CERQ; [30]).

### Heart rate variability

Heart rate was measured per beat with a telemetric heart rate monitor (Polar S810). This system allows a detection of R-R intervals with a resolution of 1 ms (i.e., sampling rate: 1000 Hz). The heart rate monitor employs an elastic electrode belt (T61, Polar Electro Oy) that is placed just below the participant’s chest muscles. The equipment was adjusted at the beginning of the experimental session. Then participants seated and rested comfortably during an acclimation phase of 10 minutes that served to monitor the quality of the recording. Data from 4 participants were discarded because of inadequate signal transmission during the experiment. Data from the remaining 35 participants with adequate signal transmission were collected during three subsequent 10 minute periods across the experiment in order to derive HRV measures before, during and after receiving the stress induction (see below).

Data were first filtered with the Polar Precision Performance Software for Windows. The errors’ detection was set at a moderate power using median and moving-average-filtering at a minimum protection zone of 6 beats per minute (see [31]). Resulting waveforms were then imported to Kubios software [32]. Samples were filtered with the low automatic filter in Kubios and visually inspected for artifacts. Kubios was then used to calculate root mean square of successive differences (RMSSD) of interbeat intervals (in milliseconds) as an index of HRV at each time period. The RMSSD has been shown to be a reliable index of cardiac parasympathetic influences [33], reflecting decreases as an effect of stress [34] and increases as an indicator of successful emotion regulation [35].

### Attentional engagement-disengagement task

#### Stimuli

Stimuli consisted of pairs of pictures comprising an emotional and a neutral facial expression of the same person. Pictures were selected from the Radboud Faces database (RaFD; [36]) based on normative data of the emotional discreteness of faces for the corresponding emotion and their valence [36]. Based on those criteria, 24 happy, disgusted and sad expressions (12 men and 12 women) for each emotional category and the corresponding neutral expressions of the same actors were selected as the stimuli. Three emotional categories were used in order to replicate previous findings using this paradigm [10], where depression-related attentional engagement and disengagement patterns were examined for happy, sad and threat-related stimuli. Unlike in the previous study [10], disgusted instead of angry faces were used as the threat category, as current research points out a greater threat perception for disgust faces (e.g., [37]). In terms of emotional discreteness, according to the validation data [36], the selected models showed a high percentage of discreteness for the proper emotions (M = 88.83, SD = 12.23). In terms of valence, disgusted and sad faces (M = 1.97, SD = .17, and M = 2.04, SD = .20, respectively) did not show significant differences (*p =* .67), whereas happy faces (M = 4.29, SD = .24) significantly differed from both (*p* = .001, in both cases).

#### Experimental design

The attention task comprised 72 trials (24 happy, 24 disgusted and 24 sad expressions paired with the corresponding neutral expression of the same actor), which were randomly presented to each participant. Emotional and neutral expressions were presented equally often on the left as on the right side of the screen. The task also included 6 practice trials, followed by a brief pause before starting the actual trials. Stimuli were displayed on a 23” screen. The size of each face was 5.8 cm (width) x 7.5 cm (height). Pictures were centered on the screen, 18 cm apart (measured from their centers). Participants were seated approximately 60 cm from the screen’s center, resulting in a visual angle of approximately 7.5 degrees between each picture’s center and the screen’s center.

**Fig 1:**
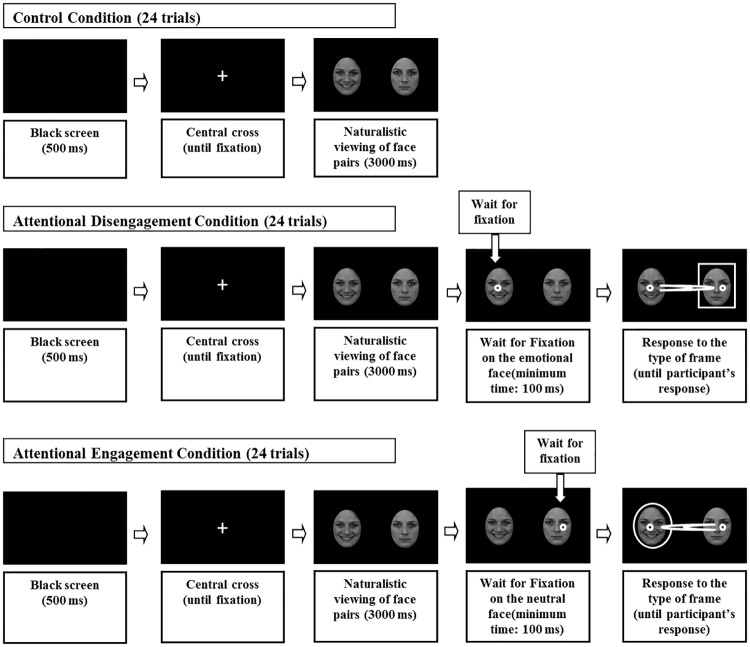
Schematic of trial presentations in the engagement-disengagement task.

The experimental design is presented in Fig 1. Each trial started with the presentation of a black screen for 500 ms, followed by the display of a white fixation cross in the middle of the screen. Immediately after the system detected a visual fixation of at least 200 ms in the cross area, a pair of faces (either happy-neutral, disgusted-neutral or sad-neutral) was presented for 3,000 ms. The engagement-disengagement procedure followed the 3,000 ms free-viewing period: 1) One third of the trials in each emotion condition (happy, disgusted, sad) assessed attentional engagement with emotional expressions. As shown in Fig 1, after the 3,000 ms free-viewing period, stimulus presentation did not continue until participants fixated on the neutral face for 100 ms. Immediately after this fixation was detected, a frame consisting of a square or a circle appeared surrounding the opposite face (i.e., emotional face). Participants were instructed to detect the frame as quickly as possible and press one of two response keys on the keyboard to indicate the type of frame (i.e., square or circle). 2) Another third of the trials assessed disengagement from emotional expressions in each emotion condition. The procedure was similar but, in this case, involving that after the 3,000 ms of free-viewing, stimulus presentation did not continue until participants fixated on the emotional face for 100 ms and then the frame appeared surrounding the opposite neutral face. 3) A last third of the trials included a regular free-viewing condition for each emotion condition, in which after the 3,000 ms free-viewing period, a new fixation cross appeared indicating the start of the next trial.

Engagement, disengagement and regular free-viewing trials for each emotional condition (i.e., happy-neutral, disgusted-neutral, sad-neutral) were randomly presented for each participant. Both types of frames in the engagement-disengagement trials were equally likely to appear in the left and right positions in all conditions. Criteria for identifying a first shift in gaze to the stimuli surrounded by the frame on each trial were identical to the ones used in previous research [10]: (a) Participants were fixated on the opposite stimulus before the frame appeared, (b) eye movements occurred at least 100 ms after the frame appeared, (c) gaze was directed to the stimulus surrounded by a frame rather than remaining at the opposite stimulus position, and d) participants made a fixation of at least 100 ms to the stimulus surrounded by a frame after shifting their gaze to it. Analyses were conducted on the resulting 93% of valid data.

#### Attention indices and reliability

The engagement-disengagement conditions employed after the 3,000 ms free-viewing period, served to establish direct measures of engagement and disengagement for each emotion condition. Indices were derived by computing the latencies from the time that the frame appeared surrounding one face (i.e., while they were fixated on the opposite one) to the time that participants made a visual fixation (100 ms) on the framed face (i.e., moved their eyes from one face to the other one and fixated on it). 1) Attentional engagement refers to the latency of that first shift in gaze from the neutral face to the emotional face surrounded by the frame in the engagement condition; and 2) attentional disengagement refers to the latency of the first shift in gaze from the emotional face to the neutral face surrounded by the frame in the disengagement condition. Reliability was examined separately for each of the six measures. The resulting Cronbach’s alpha values for valid trials within each of the six conditions were good (disengagement from disgusted: α = .75; disengagement from happy: α = .75; disengagement from sad: α = .86; engagement with disgusted: α = .77; engagement with happy: α = .74; engagement with sad: α = .80).

### Speech paradigm

#### Procedure overview

Participants were told that they would be asked to give a 5-minute speech on the topic “Why are you a good friend?”, a topic used successfully in previous studies to induce stress responses in depressed samples [10, 38]. They were told that they would have to give the speech in front of a video camera added to the eye-tracker screen and that their eye movements during the speech would be recorded. Participants were informed that their speech would be recorded and that expert psychologists would be online connected and rate their performance on clarity, coherence and persuasiveness. They were also told that they would receive feedback on their performance during the speech, consisting on the appearance of avatar faces representing evaluators’ expressions on the screen. Then the experimenter started the video connection showing the participants’ image on the screen and gave the participants two minutes to prepare the five-minute speech. The experimenter stated that during the 2-min speech preparation, he/she would go to check whether the video signal was well received in the evaluators’ rooms, and left the participant alone. After the 2 minute-preparation period, the experimenter came back to the room and stated that everyone was ready in the other rooms. Participants were then calibrated in the eye-tracker and the experimenter simulated talking by a microphone with the evaluators, by asking whether they were ready and playing voice recordings of people stating that they were ready to start with the evaluation. The overall preparation procedure lasted approximately 5 minutes. After that, participants gave the 5-minutes speech during which false feedback on their performance was shown on the screen.

#### False feedback design

The stimuli used to provide false feedback on speech performance consisted on facial expressions, representing different levels of positive and negative valence. Stimuli comprised 8 computer-generated facial models (4 male and 4 female models) created with FaceGen Modeller software (Singular Inversions, Toronto, ON, Canada). Original models were morphed using a happiness-disgusted continuum to represent different levels of feedback: accepting (expressing 100% happy), mixed (expressing 50% happy– 50% disgusted) or rejecting (expressing 100% disgusted). This resulted in 24 pictures (3 emotion labels by model) that were used to construct 7 different false feedback slides comprising the 8 false evaluators.

**Fig 2:**
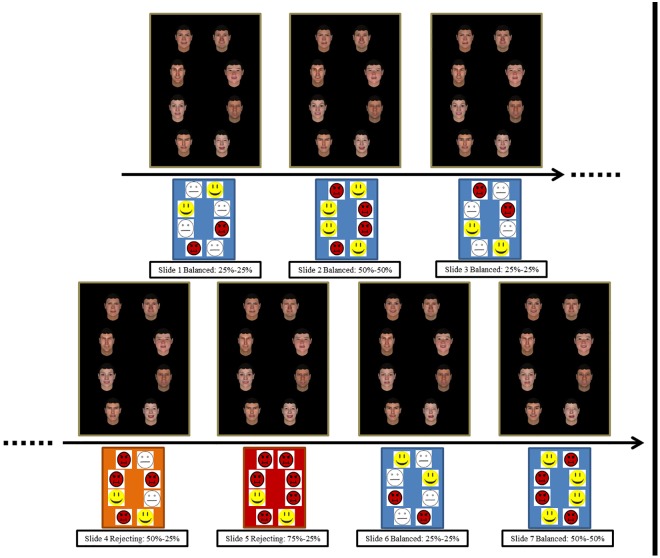
Schematic of the speech task sequence.

An overview of the false feedback procedure during the speech is shown in Fig 2. Participants were asked to focus on the screen instead of the video camera as that would help the evaluators to have a proper image of them during the video recording. An oval was presented on the center of the screen to help participants to keep focused on the screen during their speech. The oval was shown for periods of 30 sec. After each 30 sec. period, a false feedback slide appeared on the screen for 10 sec., representing the degree of acceptance/rejection of the evaluators, supposed to be online connected. This procedure (30 sec. oval– 10 sec. false feedback) was repeated 7 times, resulting in an approximate duration of 5 minutes. After the last false feedback slide, the screen turned black and participants were informed that the speech task was finished and that the video recording and the connection with the evaluators were closed.

False feedback slides comprised eight pictures (one for each model) surrounding the previous oval area. False feedback was used to separately test the effects in attention of the stress induction (attention to balanced feedback under stress) and the effects in attention to negative feedback (attention to rejecting feedback under stress) and after it (attention to balanced feedback under stress after receiving negative feedback). This was achieved by presenting false feedback slides in a fixed order: The first 2 minutes of speech involved viewing false feedback slides comprising dynamically changing balanced acceptance-rejection levels in order to test the specific effects of stress in attention (**slide 1**: 25% disgusted, 50% mixed, 25% happy; **slide 2**: 50% disgusted, 50% happy; **slide 3**: 25% disgusted, 50% mixed, 25% happy) The following 1.5 minutes of speech comprised dynamically increasing rejection over acceptance balances to test attention to rejecting feedback under stress (**slide 4**: 50% disgusted, 25% mixed, 25% happy; **slide 5**: 75% disgusted, 25% happy). The last 1.5 minutes of speech comprised again dynamically changing balanced acceptance-rejection levels in order to test attention under stress after receiving rejecting feedback (**slide 6**: 25% disgusted, 50% mixed, 25% happy; **slide 7**: 50% disgusted, 50% happy). Female and male models were distributed in the same proportion over the different screen’s positions.

#### Attention indices

The procedure involved online measurement of attention to feedback models during the speech (via eye tracking). To obtain measures of attention bias to interpersonal feedback, we considered the total fixation time (i.e., the sum of the duration across fixations) on the negative (100% disgusted models) over the positive (100% happy models) pictures in each false feedback slide. This parameter is a commonly reported index of attention bias that is sensitive to individual differences in depressive symptom severity [9, 39] and it has been used in previous impromptu speech eye-tracking paradigms [16]. A relative bias score of fixation duration to negative over positive feedback at each of the seven feedback moments (i.e., negative versus positive pictures) was calculated within-subjects, by subtracting the total fixation time on 100% happy pictures from the total fixation time on 100% disgusted pictures at each false feedback slide.

### Eye-tracker

Participants’ eye movements in both tasks were recorded using a Tobii TX300 eye-tracker system. This system employs a dual-Purkinje eye-tracking method [40] and samples eye-gaze coordinates at 300 Hz (e.g., a coordinates’ estimation every 3.3 ms). A 9-point grid calibration procedure was completed before performing each of the tasks. Both stimuli presentation and eye movements’ recording were controlled by E-prime Professional software [41]. The eye-tracking system synchronized automatically with each of the two programs at the start of each trial/false feedback slide. Eye movement signals were converted to visual fixation data by using E-prime extensions for Tobii (i.e., Clearview PackageCalls).

### Feedback pictures’ rating task

Participants also completed a rating task in order to validate emotional characteristics of the pictures employed in the stress induction paradigm (false feedback pictures). Pictures were shown on the computer screen, one by one, and participants were asked to identify and rate the emotion expressed by the face. Each trial began with a central fixation cross presented for 500 ms. The picture (5.8 x 7.5 cm) was then presented along with three categories: 1 –disgusted, 2 –not disgusted nor happy, 3 –happy. Participants then selected which category best described the emotion expressed in the picture by pressing the appropriate key. This served to evaluate the discreteness’ degree of each picture to the emotion depicted (100% disgusted, 50% disgusted– 50% happy, or 100% happy). After that, the picture remained on the screen, and participants were presented with a line with nine labeled anchor points, ranging from 1 (very negative) to 9 (very positive). This scale served to evaluate the position in the negative-positive valence dimension for each picture. Once participants rated the picture by pressing the appropriate key, the next trial began. The rating task comprised a total of 24 trials.

In terms of discreteness’ to the emotion depicted, 100% disgusted and 100% happy computer-generated faces were adequately assigned to their given emotional category, 90% and 93% of times, respectively. Regarding to 50% disgusted– 50% happy mixed faces, they were assigned to the intermediate emotional category (not disgusted nor happy) 37% of times, whereas they were identified as happy faces the 52% of times, and as disgusted faces the remaining 11% of times. The latter results confirm the mixed emotional nature of the stimuli. However, in terms of dimensional valence, the three picture conditions significantly differed in the expected directions, *F*(2,36) = 519.01, *p =* .001, _p_^2^ *=* .97, with the 100% disgusted models (M = 2.74, SD = 0.62) identified as significantly more negative than both the 50%-50% mixed models (M = 5.52, SD = 0.47) and the 100% happy models (M = 7.16, SD = 0.53), and the 100% happy models identified as significantly more positive than the 50%-50% mixed models, all *p’s =* .001.

### Design overview

**Fig 3:**
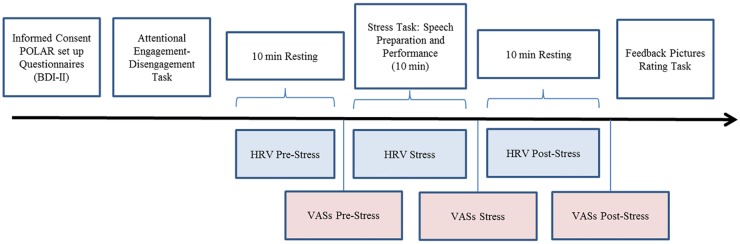
Schematic of the tasks sequence during the session.

Fig 3 depicts the sequence of tasks. Participants were told at the beginning of the experimental session that the study would involve the evaluation of eye-movements during different tasks of emotional perception and expression. Participants gave their written informed consent, the polar equipment was put on, and then they completed a series of questionnaires, including the BDI-II. Thereafter participants completed the attentional engagement-disengagement task. After completing this task, they remained seated and rested for 10 minutes (pre-stress HRV measure), and then rated their current mood state (VASs pre-stress mood). Then they received the instructions for the stress task (3 minutes) and prepared their speech for an extra 2 minutes. After that they gave the 5-min speech during which their gaze behavior to false feedback was monitored and recorded. The overall stress manipulation procedure (stress anticipation and speech) lasted for approximately 10 minutes (stress reactivity HRV measure). Immediately after finishing the speech they rated again their current mood state (VASs stress-mood). Participants sat and rested then for another 10 minutes (post-stress recovery HRV measure), and after this recovery period, they rated again their current mood state (VASs post-stress mood). The session finished with the feedback pictures’ rating task. At the end of the procedure participants were fully debriefed on the real purposes of the study and completed a brief questionnaire to test the level of suspiciousness on the real aims of the stress induction procedure. Finally, they received a brief positive mood induction aimed to reduce negative mood level [42] and were thanked and compensated for their participation. The experimental session lasted approximately 90 min.

### Statistical analyses

The data-analytic strategy comprised three steps. First, we examined the relationship between depressive symptom severity and attention biases under standard (attentional engagement-disengagement task) and during stress conditions (attention biases to false feedback during the speech), as well as with differences in self-reported (VASs changes) and physiological (HRV changes) responses as result of the stress induction. To increase the statistical power of our analyses, this was performed by conducting a series of mixed-design analyses of covariance (ANCOVAs) for each measure, introducing depressive symptom severity level as a covariate. When the covariate accounted for any of the main or interaction effects, bivariate correlations between depressive symptom severity level and the corresponding indices were performed to clarify the direction of the associations. Further analyses using a group-level approach were also performed, comprising Bonferroni-corrected post-hoc comparisons between dysphoric (n = 15) and non-dysphoric (n = 24) participants (according to BDI-II cut-off score = 14; [26]), in order to further clarify group level differences at those dependent variables where depressive symptom severity was identified as a significant covariate.

Secondly, we examined whether standard measures of depression-related attention bias (attentional engagement-disengagement biases) were associated with attentional bias under stressful socio-evaluative conditions. Bivariate correlations between attention bias measures in the attentional engagement-disengagement task and attention bias measures in the speech procedure were performed for those indices where depressive symptom severity level acted as a significant covariate (i.e., associations between depression-related attention biases at standard and stress conditions).

Finally, we tested mediational models examining the predictive role of depressive symptom severity level in stress reactivity and stress regulation responses via its association with depression-related attention biases. Stress reactivity and stress regulation measures were computed for those self-reported (VASs) and physiological (HRV) indicators where effects of the stress induction were found. Stress reactivity/regulation scores were constructed using simple linear regression models in which scores at a given time were predicted by their corresponding previous scores (e.g., physiological reactivity: HRV during the pre-stress period predicting HRV during the stress period; physiological recovery: HRV during the stress period predicting HRV during the post-stress period), and the resulting standardized residuals were saved. Standardized residuals control for variability in baseline scores and are considered a reliable method to compute changes in stress level (e.g., [10, 43]). Bivariate correlations between observed depression-related attention biases and stress reactivity/recovery measures were examined. When significant associations between depression-related attention biases and stress changes were found, mediational models were used to examine total effects (i.e., effect of depressive symptom severity on stress change without taking into account attention bias; path *c*), direct effects (i.e., effect of depressive symptom severity on stress change after controlling for attention bias; path *c’*), and indirect effects in those associations (i.e., effect of depressive symptom severity on stress change via attention bias; path *a* × *b*). Mediation models were constructed using a bootstrapping approach [44]. By relying on confidence intervals to determine the significance of the indirect effect, this statistical method avoids problems associated with traditional approaches (e.g., unrealistic assumptions regarding multivariate normality) (see [45]). The estimated 5000 bias-corrected bootstrap 95% confidence intervals should not contain zero to be significant [44].

## Results

### Attention biases under natural conditions

Mean and standard deviations for each attention bias index for the whole sample are summarized in Table 1.

**Table 1:** Mean and standard deviations of attention bias measures in the study.

	M	SD
Attentional Disengagement (sec)		
Disgusted	0.29	0.07
Happy	0.28	0.05
Sad	0.29	0.08
Attentional Engagement (sec)		
Disgusted	0.30	0.09
Happy	0.28	0.05
Sad	0.30	0.09
Fixation Duration to Negative over Positive Pictures during the Speech (diff in sec)		
Overall (full speech)	0.38	0.50
Slide 1 (balanced feedback)	-0.19	1.13
Slide 2 (balanced feedback)	-0.13	1.04
Slide 3 (balanced feedback)	0.26	1.21
Slide 4 (rejecting feedback)	0.98	1.53
Slide 5 (rejecting feedback)	1.50	1.71
Slide 6 (balanced feedback)	0.26	0.88
Slide 7 (balanced feedback)	-0.15	1.69

*Notes*. M = mean; SD = Standard Deviation; diff = difference; sec = seconds

#### Attentional disengagement

**Fig 4:**
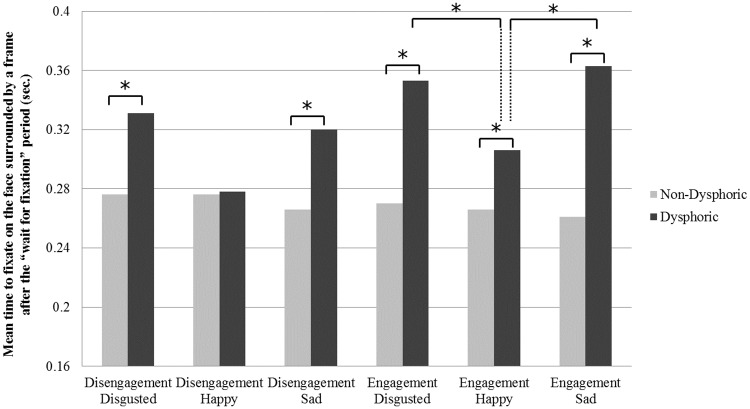
Mean times to direct attention to the face surrounded by a frame in the attentional engagement and disengagement conditions for each emotion condition comparing non-dysphoric and dysphoric participants. * *p <* .05

The mixed design ANCOVA with emotion (happy, disgusted, sad) as within-subject factors and depressive symptom severity as covariate revealed a near to significant main effect of emotion, *F*(2,36) = 3.21, *p* = .05, η^2^ = .15, which was accounted by a significant interaction of emotion by depressive symptom severity, *F*(2,36) = 6.57, *p* = .004, η_p_^2^ = .27. To further investigate the interaction effect, bivariate correlation coefficients between depressive symptom severity and the three attentional disengagement indices (i.e., happy, disgusted and sad stimuli) were calculated. Analyses showed that higher depression severity levels were significantly associated with longer times to disengage attention from both disgusted, *r* = .42, *p* = .008, and sad faces, *r* = .56, *p* = .001, whereas the correlation with attentional disengagement from happy faces was not significant, *r* = .04, *p* = .80. Bonferroni corrected post-hoc comparisons between dysphoric and non-dysphoric participants showed that such associations were explained by significantly longer times to disengage attention from both disgusted and sad faces in the dysphoric compared to the non-dysphoric group, *p* = .02 and *p* = .04, whereas groups did not differ in their time to disengage attention from happy faces, *p* = .89 (see Fig 4).

#### Attentional engagement

The mixed design ANCOVA with emotion (happy, disgusted, sad) as within-subject factors and depressive symptom severity as covariate revealed a significant main effect of depressive symptom severity, *F*(1,37) = 17.80, *p* = .001, η_p_^2^ = .33, which was accounted by a significant interaction of emotion by depressive symptom severity, *F*(2,36) = 3.57, *p* = .039, η_p_^2^ = .17. Bivariate correlation showed that higher depression severity levels were significantly associated with longer times to engage attention with all types of emotional faces, happy, *r* = .42, *p* = .007, disgusted, *r* = .51, *p* = .001, and sad faces, *r* = .57, *p* = .001. Bonferroni corrected post-hoc comparisons between dysphoric and non-dysphoric participants confirmed significantly longer times to engage attention with happy, disgusted and sad faces in the dysphoric compared to the non-dysphoric group, *p* = .021, *p* = .005 and *p* = .001, respectively. However, when attentional engagement indices were compared within-groups, whereas no differences were found in the non-dysphoric group, all *p’s* > .05, the times to engage attention towards disgusted and sad faces were significantly longer than the times to engage attention towards happy faces in the dysphoric group, *p* = .009, and *p* = .049, respectively (see Fig 4). Therefore, although higher depressive symptom severity was associated with a general longer time of attentional engagement with all emotional information, this association was significantly higher for attentional engagement with negative information (i.e., disgusted and sad faces).

### Attention biases under stress conditions

Mean and standard deviations for attention bias indices (fixation duration to negative over positive pictures) during the speech for the whole sample are summarized in Table 1.

**Fig 5:**
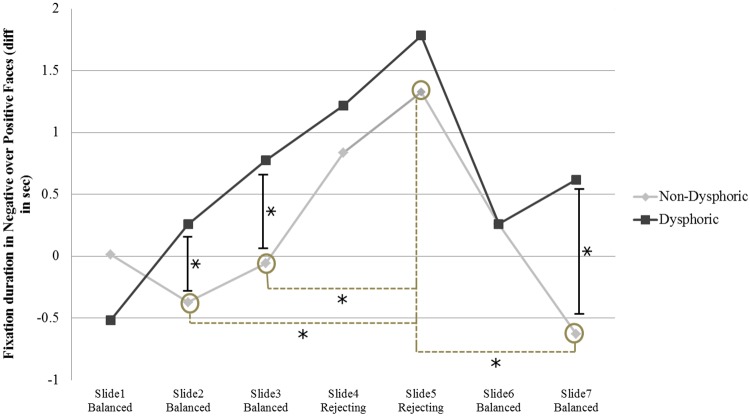
Fixation duration in negative over positive faces for each feedback slide during the speech comparing non-dysphoric and dysphoric participants. * in solid lines = between-group differences *p <* .05; * in broken lines = within-group differences *p <* .05; diff in sec = difference in seconds.

A mixed design ANCOVA was conducted to analyze attention bias across the speech, with time (the seven false feedback slides) as within-subject factor and depressive symptom severity as covariate. First, analyses revealed a main effect of time, *F*(6,32) = 4.54, *p* = .002, η_p_^2^ = .46, explained by longer fixation duration to negative over positive pictures in the false feedback slides comprising rejecting feedback (i.e., slides 4 and 5) in comparison to those comprising balanced feedback presented before (i.e., slides 1, 2 and 3) and after (i.e., slides 6 and 7), all *p’s <* .05. Second, analyses revealed both a significant main effect of depressive symptom severity, *F*(1,37) = 6.86, *p* = .013, η_p_^2^ = .16, as well as a significant depressive symptom severity by time interaction, *F*(6,32) = 3.38, *p* = .011, η_p_^2^ = .38. Bivariate correlation showed that higher depression severity levels were associated with an overall longer fixation duration to negative over positive pictures across the speech, *r* = .50, *p* = .001 In order to clarify this general effect, correlations were separately examined for each of the seven false feedback slides, showing that higher depression severity scores were associated with longer fixation duration to negative over positive pictures in balanced feedback slides presented both before and after rejecting feedback slides (slide 3: *r* = .31, *p* = .049, slide 7: *r* = .58, *p* = .001) whereas no significant associations were found for rejecting feedback slides presented in between (slide 4: *r* = .12, *p* = .49, slide 5: *r* = .01, *p* = .96). Bonferroni corrected post-hoc comparisons between dysphoric and non-dysphoric participants confirmed significant differences between groups: Before being presented with rejecting feedback slides (4 and 5), dysphoric participants were characterized by longer fixation duration to negative over positive pictures in balanced feedback slides (slide 2: *p* = .05, slide 3: *p* = .04). Then, groups did not differ in their fixation duration when rejecting feedback slides were presented (slide 4: *p* = .46, slide 5: *p* = .43). Groups differed again in their fixation duration to balanced feedback slides after having being presented with rejecting feedback, with dysphoric participants being characterized by longer fixation duration to negative over positive pictures in slide 7, *p* = .023. No other between-group comparisons reached significance. Importantly, when within-group comparisons were performed, non-dysphoric individuals showed shorter fixation duration to negative over positive pictures in balanced feedback slides presented both before (slides 2 and 3) and after (slide 7) rejecting feedback (slide 5), all *p’s >* .05. In contrast, fixation duration to negative over positive pictures did not differ between false feedback slides across the whole speech in the dysphoric group, all *p’s <* .05 (see Fig 5). Overall, results showed an association between higher depression severity and longer times attending to negative over positive pictures across the whole speech. This effect was qualified by different patterns of attention: Whereas non-dysphoric individuals were characterized by a balanced processing of positive and negative pictures across the speech, only showing a longer fixation duration to negative over positive pictures when received clearly rejecting feedback (slides 4 and 5), dysphoric individuals showed this biased fixation duration pattern across the whole speech.

### Associations between attention biases at different conditions

Bivariate correlations were conducted to analyze the association between depression-related attention biases found under standard conditions (attentional disengagement and engagement biases for disgusted and sad faces) and depression-related attention biases under social stress (i.e., overall fixation duration to negative over positive pictures across the whole speech, as well as for those false feedback slides where significant effects were found).

Correlations are summarized in Table 2. Higher overall fixation duration to negative over positive feedback across the speech was associated with longer times to disengage attention from sad faces and with longer times to engage attention with both types of negative faces. When specific false feedback slides were analyzed, this general association was accounted by both types of depression-related attention biases: longer times to both disengage attention from and engage attention with negative faces were significantly associated with longer fixation duration to negative over positive pictures after receiving rejecting feedback (i.e., slide 7). In order to clarify whether depression-related attention biases would predict differential attention patterns from rejecting to balanced feedback in the speech, changes in attention bias duration from rejecting feedback (slide 5) to balanced feedback (slide 7) were computed. Correlation analyses showed that longer times to both disengage attention from and engage attention with negative faces in standard conditions were associated with higher sustained attention to negative over positive pictures (i.e., less fixation duration decrease) from rejecting to balanced feedback, all *r’s >* .34, all *p’s <* .05.

**Table 2:** Correlation between depression-related attention bias indices.

	Attentional Disengagement Disgusted	Attentional Disengagement Sad	Attentional Engagement Disgusted	Attentional Engagement Sad
Fixation Duration to Negative over Positive Pictures during the Speech (diff in ms)				
Overall (full speech)	.20	.31*	.45**	.50**
Slide 1 (balanced feedback)	-.33*	-.17	-.14	-.09
Slide 2 (balanced feedback)	.10	-.05	.27	.19
Slide 3 (balanced feedback)	-.20	-.08	.17	.26
Slide 4 (rejecting feedback)	.03	-.01	.06	.08
Slide 5 (rejecting feedback)	.13	.10	.13	.24
Slide 6 (balanced feedback)	.15	.16	.22	.27
Slide 7 (balanced feedback)	.34*	.41**	.38*	.28

Notes.

* *p<* .05;

** *p<* .01;

### Speech effects in stress reactivity and recovery

Mean and standard deviations for both self-reported mood and HRV scores are summarized in Table 3.

**Table 3:** Mean and standard deviations of stress reactivity and recovery measures in the study.

	M	SD
Self-reported Happy mood		
Pre-Stress	2.92	2.57
Stress	3.59	2.31
Post-Stress	3.31	2.45
Self-reported Sad mood		
Pre-Stress	1.28	2.03
Stress	1.00	1.45
Post-Stress	1.03	1.71
Self-reported Tense mood		
Pre-Stress	1.72	1.99
Stress	4.05	2.46
Post-Stress	2.38	2.20
HRV (RMSSD)		
Pre-Stress	35.67	20.15
Stress	28.22	14.80
Post-Stress	33.39	19.95

*Notes*. M = Mean, SD: Standard Deviation

#### Self-reported stress reactivity and recovery

A 3 x 3 mixed design MANCOVA was used to examine speech effects in VASs scores, with mood state (happiness, sadness, tension) and assessment time (Time 1: pre-stress, Time 2: during-stress, Time 3: post-stress) as within-subject factors, and depressive symptom severity as covariate. Analyses revealed a significant two-way mood state by assessment time interaction, *F*(4,34) = 5.64, *p* = .001, η_p_^2^ = .40, not accounted by depressive symptom severity, *F*(4,34) = 0.13, *p* = .97, η_p_^2^ = .01. Consequently, separate analyses were conducted for each mood state. No significant main effects of time were found either for happiness or sadness, *F*(2,36) = .85, *p* = .43, η_p_^2^ = .04, and *F*(2,36) = .51, *p* = .64, η_p_^2^ = .03, respectively. For tension, the main effect of time was significant, *F*(2,36) = 13.18, *p* = .001, η_p_^2^ = .42. Bonferroni post-hoc comparisons showed that there was a significant increase in the tension level from the pre-stress to the stress period, *p =* .001, and by a subsequent decrease in the tension level from the stress to the post-stress period, *p =* .001. These main effects were not qualified by a significant time by depressive symptom severity interaction, *F*(2,36) = 0.46, *p* = .63, η_p_^2^ = .02.

#### Physiological stress reactivity and recovery

A mixed design ANCOVA with assessment time (Time 1: pre-stress, Time 2: during-stress, Time 3: post-stress) as within-subject factor, and depressive symptom severity as covariate was used to test changes in HRV. The main effect of assessment time was significant, *F*(2,32) = 5.48, *p* = .009, η_p_^2^ = .26. Bonferroni post-hoc comparisons showed that there was a significant decrease in HRV from the pre-stress to the stress period, *p =* .001, and by a subsequent increase in HRV from the stress to the post-stress period, *p =* .033. These main effects were not qualified by a significant time by depressive symptom severity interaction, *F*(2,32) = 2.18, *p* = .13, η_p_^2^ = .12.

### Predictors of stress reactivity and recovery

We examined whether individual differences in depression-related attention biases found under natural and stress conditions were associated with self-reported (i.e., tension) and physiological (i.e., HRV) changes observed in response to the stressor (stress reactivity) and/or during the subsequent recovery period (stress recovery). No associations were found for self-reported tension changes from the pre-stress to the stress period, all *r’s <* 0.22, all *p’s >* .05. However, all depression-related attention biases under standard conditions (i.e., attentional disengagement from and attentional engagement with both disgusted and sad faces) were positively associated with self-reported tension changes from the stress to the post-stress period, all *r’s >* 0.41, all *p’s <* .01. Furthermore, a similar significant association was found for overall fixation duration to negative over positive pictures across the speech and subsequent self-reported tension changes from the stress to the post-stress period, *r =* 0.48, *p =* .002. Regarding changes in HRV, no significant associations were observed in changes neither from the pre-stress to the stress period, all *r’s <* 0.16, all *p’s >* .05, nor from the stress to the post-stress period, all *r’s <* 0.29, all *p’s >* .05.

Mediational models were used to test the indirect effect of depressive symptom severity levels (predictor) in self-reported stress recovery (tension changes from the stress to the post-stress period; outcome) via their association with attention biases associated to that change (attentional disengagement from and attentional engagement with both disgusted and sad faces, and overall fixation duration to negative over positive pictures across the speech; mediators). Mediation models are summarized in Table 4. Analyses showed that neither the total effect (i.e., effect of depressive symptom severity on self-reported stress recovery without taking into account attention biases; path *c*), nor the direct effects (i.e., effect of depressive symptom severity on self-reported stress recovery after controlling for the corresponding attention bias; path *c’*) were significant. However, the indirect effect of each model (i.e., effect of depressive symptom severity on self-reported stress recovery via the corresponding attention bias; path *a* × *b*) was significant. Therefore, higher depressive symptom severity levels were indirectly associated with more positive change scores in the self-reported change measure (i.e., less stress recovery) via their associations with 1) longer attentional disengagement from negative faces, 2) longer attentional engagement with negative faces, and 3) longer fixation duration to negative over positive pictures during the speech.

**Table 4:** Serial mediational models tested.

Independent Variable (IV)	Mediator (M)	Dependent Variable (DV)	Total effect (c)	Direct effect *(c´)*	Indirect effect	
					(*a* x *b*)	95% CI
Depressive symptom severity	Attentional disengagement disgusted faces	Self-reported stress recovery (tension level change)	.04 (SE = .01)	.03 (SE = .02)	.01 (SE = .01)	(.0006 to .0352)
Depressive symptom severity	Attentional disengagement sad faces	Self-reported stress recovery (tension level change)	.04 (SE = .01)	.02 (SE = .02)	.01 (SE = .01)	(.0001 to .0476)
Depressive symptom severity	Attentional engagement disgusted faces	Self-reported stress recovery (tension level change)	.04 (SE = .01)	.01 (SE = .01)	.02 (SE = .01)	(.0079 to. 0485)
Depressive symptom severity	Attentional engagement sad faces	Self-reported stress recovery (tension level change)	.04 (SE = .01)	.01 (SE = .02)	.03 (SE = .01)	(.0100 to. 0530)
Depressive symptom severity	Overall fixation duration to negative over positive pictures during speech	Self-reported stress recovery (tension level change)	.04 (SE = .01)	.02 (SE = .02)	.02 (SE = .01)	(.0026 to. 0459)

*Notes*. SE = Standard error

### Further analyses: Suspiciousness during the speech

Regarding to whether participants suspected that they were not being video recorded during the speech, a 20.5% reported suspiciousness about this issue. Regarding to whether participants suspected that there were no evaluators online connected during their speech, a 35.9% reported suspiciousness about it. Further analyses were conducted comparing participants who reported any type of suspiciousness (n = 14) and participants who did not report any suspiciousness on the false feedback design (n = 25). Analyses showed that groups did not show different patterns of sustained attention to negative over positive feedback during the speech, *F*(1,37) = 0.61, *p =* .44, η_p_^2^ *=* .02, and were not characterized by different patterns of change either in self-reported stress, *F*(2,36) = 0.08, *p =* .92, η_p_^2^ *=* .01, or HRV measures, *F*(2,32) = 0.49, *p =* .62, η_p_^2^ *=* .03, across the stress induction procedure. Therefore, the degree of suspiciousness did not differently affect to responses in the stress induction procedure observed in the main analyses.

## Discussion

Attention biases during natural viewing of social information (i.e., sustained attention to negative faces as the result of difficulties disengaging from them [8] are thought to be key aspects in depression development and maintenance [6]. However, little is known on the specific conditions where such mechanisms operate (e.g., whether these depression-related attention biases emerge under socially stressful situations, such as giving a speech). The present study was aimed to clarify: 1) the presence of depression-related attention bias related to a social stressor, 2) its association with depression-related difficulties disengaging attention from negative faces as measured under standard conditions, and 3) the association of these maladaptive attention processes with stress reactivity and recovery.

Our design involved the assessment of attention biases to emotional faces during standard conditions, using a previously validated eye-tracking paradigm (the attentional engagement-disengagement task; [10]), which was followed by an impromptu speech where eye-tracking was used to monitor gaze patterns towards social feedback during a stressful socio-evaluative situation. Results with the attentional-disengagement task replicate previous findings [10]. Higher depression severity levels were associated with longer times to move gaze away from negative faces when participants were prompted to engage with an opposite neutral counterpart. This effect was similar to the one previously reported [10], where clinically depressed compared to control individuals were characterized by longer times to disengage their attention from sad faces. Of note, whereas the previous study found evidence of this attentional disengagement bias for sad but not angry faces [10], in the present study we found support for an association between higher depression severity levels and longer attentional disengagement biases from both sad and disgusted faces. Although both angry and disgusted expressions comprise a threatening signal with social meaning (“social disapproval”), research has shown that disgusted faces may comprise a greater social-threat perception than angry faces (e.g., [37]). The fact that depression level was associated with attentional disengagement from mood-congruent (sad faces) but also with social disapproval signals (disgusted faces) provides initial support for the prediction that depression-related attention biases may be at the basis of difficulties in social interactions (e.g., [11]).

Furthermore, higher depression severity levels were also associated with longer times to move gaze away from neutral faces when prompted to engage with emotional counterparts, and this bias was especially pronounced for negative faces (i.e., delayed engagement with both sad and disgusted faces). Given the nature of this paradigm (engagement-disengagement patterns after 3,000 ms of free viewing), slower attentional engagement with emotional faces observed in this study might reflect a volitional avoidance of processing of emotional information once it has already been processed rather than a deficit in the attentional capture by emotional features. Further research is necessary to clarify the nature of this pattern. Future studies should test whether delayed attentional engagement patterns specifically emerge under late-processing conditions (after 3,000 ms of free viewing) in contrast to early-processing conditions (initial engagement patterns when emotional faces are presented).

We aimed to clarify whether depression-related attention bias would also emerge under specific stressful socio-evaluative conditions. First, our findings in the impromptu speech support an association between higher depression severity levels and sustained attention to negative social signals across the speech. In line with recent research using this eye-tracking based paradigm [16], maladaptive attention processing of social information during the socio-evaluative condition was evidenced by longer times attending to negative than to positive pictures in the audience. Our study is the first to examine this association in depression, suggesting that this can be a common pattern of processing in problems associated to difficulties in social interactions. Furthermore, the paradigm employed in our study had the advantage of presenting social feedback in dynamic change, which may better address the way attentional processing is driven during real social interactions [17]. The specific dynamic change pattern employed in our paradigm (i.e., balanced-rejecting-balanced feedback) allowed disentangling specific contexts were depression-related attention biases emerged. Whereas depression severity level was associated with longer fixation duration to negative over positive pictures during balanced feedback contexts (similar to results observed in other social interaction problems; [15, 16]), when feedback changed to a clearly rejecting audience, participants across all depression severity levels were characterized by sustained attention to negative over positive pictures. Post-hoc comparisons clarified this pattern by showing that whereas dysphoric individuals were characterized by a fixed pattern of sustained attention to negative over positive pictures across the speech, non-dysphoric individuals showed a flexible pattern of processing: balanced attentional processing of negative and positive pictures when receiving balanced feedback, but increases of attention towards negative pictures when the audience increased its level of social rejection. These findings suggest that adaptive processing of social information may be characterized by flexible attention towards negative and positive social signals, as a function of contextual changes. In contrast, individuals at high levels of depression severity would be characterized by inflexible patterns of processing, not adjusting their processing of social signals to the contextual demands. This pattern was directly visible by changes in attention from rejecting feedback (slide 5) to subsequent balanced feedback (slide 7): whereas non-dysphoric participants adjusted their attentional processing, reducing their attention to negative pictures in the following balanced context, dysphoric participants continued showing sustained attention to negative pictures, regardless the change in the social context.

The second aim of the study was to examine whether depression-related attention biases assessed during standard conditions (delayed engagement with social information and difficulties disengaging from negative social signals when attention is captured by them) would be associated with specific attentional processing under stressful socio-evaluative conditions. Our results indicate that both depression-related delayed disengagement from and engagement with negative faces were associated with sustained attention in negative over positive pictures across the speech. Importantly, when specific dynamic changes during the speech were taken into account, patterns of both delayed engagement with and disengagement from negative faces were associated with more sustained attention to negative over positive pictures after receiving rejecting feedback (slide 7). Depression-related difficulties disengaging attention from negative faces were also associated with sustained attention to negative pictures when social feedback changed from rejecting to balanced (from slide 5 to slide 7), indicating that participants characterized by attentional disengagement difficulties under standard conditions also showed less flexible patterns in adjusting their attention to the contextual change in the socio-evaluative situation.

Taken together, these findings may help to contextualize attentional mechanisms during social interactions, elucidating the mechanisms behind enduring negative views of oneself and the situation in depressed individuals. Depressed individuals tend to perceive social interactions as negative and attribute negative outcomes derived from them to themselves [46, 47]. The subjective experience of social interactions in depressed patients has been characterized by a diminished desire to socially interact and a common experience of fear to be involved in them [48]. The presence of depression-related biases comprising both delayed engagement with social information, but also delayed disengagement when attention is captured by negative signals, may not only be explained by these motivational states, but may also further intensify these negative cognitions. First, if further research is able to replicate the presence of depression-related delayed engagement with social information (emotional faces) this bias could be reflecting a mechanism of avoidance to engage in social interactions. Based on significant correlations found in our study, this avoidance mechanism might result in a sustained processing of negative feedback in depressed individuals when they cannot avoid being involved in those situations (fixation duration to negative feedback across the speech). A second mechanism comprising difficulties to disengage attention from negative social information when it is attended might help to explain inflexible processing of social feedback during dynamic interactions. The fact that disengagement biases were associated with reduced regulation of attention from clearly rejecting to subsequent balanced socio-evaluative feedbacks in the speech suggests: 1) that depressed individuals may experience difficulties disengaging attention from negative signals during socio-evaluative conditions in a similar way that they do during natural processing conditions, and 2) that this impairment may emerge when regulation of attention resources is required (inhibit processing of negative social signals when the context changes to a more balanced social feedback). Although these results are preliminary and require further replication, they may help to identify different mechanisms by which depressed individuals would experience sustained negative affect and post-event ruminations after being involved in stressful social interactions (e.g., [12]).

The last aim of the study was to replicate and extend previous evidence on the association of depression-related attention biases with inefficient regulation of social stress responses [10]. Replicating previous findings, mediation analyses showed that depression-related biases in the disengagement from negative faces were related to higher stress levels during the recovery phase (i.e., poorer stress regulation after the stressful socio-evaluative situation). This finding is also congruent with previous results supporting an association between impaired attentional disengagement and changes in negative mood in other stress induction paradigms [49, 50]. Furthermore, a similar mediational model was supported for depression-related biases in the engagement with negative faces, indicating that both attention mechanisms may contribute to sustained stress levels in depression. Importantly, when the role of fixation duration to negative over positive feedback during the speech was considered in the model, a similar mediation model was supported. Overall, these results indicate that higher depression severity levels were associated with sustained stress levels via their associations with 1) longer attentional disengagement from negative faces, 2) longer attentional engagement with negative faces, and 3) longer sustained attention to negative over positive pictures during the speech. Delayed engagement with social information and difficulties disengaging from negative signals when they are attended may preclude depressed people from using an effective attention processing when confronted with social interactions (flexible dynamic changes in attention during the speech, as observed in non-dysphoric participants), resulting in sustained processing of negative information. In turn, inflexible processing of negative social signals might interfere with the ability to successfully reframe negative situations using reappraisal and contribute to the continuous post-processing of negative information observed in depressed individuals, such as rumination [51], leading to prolonged negative affect [6].

On an exploratory basis, we also examined whether such associations would be replicated using objective physiological indictors of stress regulation (HRV). Although the speech task has been effective on inducing HRV decreases as an effect of stress [34] and HRV increases as an indicator of successful emotion regulation during recovery [35] in our sample, we did not find evidence for significant direct associations between depression severity level and HRV changes. In a similar vein, other studies with analogue samples have failed to find differences between dysphoric and non-dysphoric individuals in HRV responses to stress (e.g., [52]), whereas depression level is found to be a predictor of dysfunctional HRV in response to stress in clinical samples (e.g., [53]). Further research will require testing the association of depression-related attention biases with objective measures of stress regulation (i.e., HRV increases) in clinically depressed samples. Furthermore, the non-clinical nature of the recruited sample and the assessment of depressive symptom severity via a self-report measure limit to some extent the generalizability of our findings. Further investigation of attention processes under dynamic socio-evaluative conditions, its connection with depression-related attention biases under standard conditions, and its role in stress regulation processes is needed across different samples representing the depression course (i.e., samples of non-depressed at-risk, clinically depressed, and formerly depressed individuals). The cross-sectional nature of our design also precludes causal conclusions regarding the assumed influence of depression-related attention biases on gaze behavior during socio-evaluative conditions as well as on the role of those biased attention patterns in stress regulation. Direct proofs of cause-and-effect relations will require experimental manipulation of attention biases (e.g., via attention training) to examine training-related changes in attention behavior to social feedback and in the regulation of social stress responses.

To summarize, the novel eye-tracking paradigms we used allowed for the continuous monitoring and direct estimations of visual attention processes involved in the processing of social information. This methodology holds potential to increase our insight into the potential involvement of attention biases in social difficulties observed in depression, suggesting that depression-related biases emerge under both standard and socio-evaluative conditions, and that they may share a common predictive role in emotional dysregulation of social stress.

## Supporting information

S1 File Dataset. Original data set of all measurements mentioned in this manuscript.
(SAV)
